# Advances in Photothermal Electrospinning: From Fiber Fabrication to Biomedical Application

**DOI:** 10.3390/polym17131725

**Published:** 2025-06-20

**Authors:** Jingwen Liu, Kai Wang, Fengying Jin, Yile Bin, Jiayi Li, Xiaofei Qian

**Affiliations:** 1 The First School of Clinical Medicine, Southern Medical University, Guangzhou 510515, China; 2 School of Microelectronics, Fudan University, Shanghai 200433, China

**Keywords:** electrospinning, photothermal therapy, photothermal agents, near-infrared irradiation, antibacterial, anticancer, tissue regeneration

## Abstract

Photothermal electrospinning (PTE) represents an innovative fusion of electrospinning (ES) technology and photothermal therapy (PTT), where photothermal agents (PTAs) are incorporated into electrospun fibers to enable localized thermal effects under near-infrared (NIR) irradiation. The high surface area and tunable architecture of electrospun fibers provide an ideal platform for efficient PTA loading, while the precise temperature control and therapeutic efficacy of PTT significantly broaden its biomedical applications, including antibacterial therapy, anticancer treatment, tissue regeneration, and drug delivery. This review mainly focuses on the emerging field of PTE. Following an overview of the basic PTE parts (ES, PTAs, and PTT), the fabrication strategies (one- and two-step methods) of photothermal electrospun fibers and their latest advancements in both antibacterial and non-antibacterial applications are summarized. Furthermore, the current challenges are deliberated at the end of this review.

## 1. Introduction

Electrospinning technology, as an advanced method for efficient fabrication of micro- and nanoscale fibers, can be traced back to the 17th century. Since the late 20th century, it has garnered significant attention in the materials science and biomedical fields [1,2]. This technique employs high-voltage electric fields to stretch polymer solutions or to melt them into continuous fibers with diameters ranging from tens of nanometers to several micrometers. The resulting fibrous materials possess unique advantages, including their high specific surface area, tunable porosity, and exceptional structural design flexibility [3,4,5,6]. In recent years, with progress in nanotechnology and functional materials, electrospinning has evolved from a simple fiber production technique to a versatile platform for developing multifunctional materials, demonstrating tremendous potential, particularly in biomedical applications [7,8,9,10,11].

Photothermal therapy (PTT) has emerged as a novel treatment modality that utilizes photothermal agents (PTAs) to convert light energy into localized thermal energy, exhibiting remarkable advantages in antibacterial therapy, tumor treatment, tissue repair, and drug delivery. Compared with conventional therapeutic approaches, PTT offers distinct benefits, including non-invasiveness, spatiotemporal controllability, and low systemic toxicity. However, traditional PTT techniques face several challenges such as insufficient targeting capability of PTAs, thermal diffusion-induced damage to healthy tissues, and limited therapeutic efficacy [12,13,14,15]. To address these limitations, researchers have begun exploring the integration of PTAs with electrospinning technology to develop novel electrospun materials with photothermal conversion capabilities.

Photothermal electrospinning (PTE) technology, building upon the basic electrospinning (ES) process, achieves the innovative integration of materials science and photothermal therapy by incorporating PTAs either during or after fiber fabrication [16,17,18,19]. This approach not only preserves the structural advantages of electrospun fibers but also endows the materials with photoresponsive characteristics, providing novel solutions for precision medicine. PTE technology offers several significant advantages: (i) the three-dimensional porous structure of electrospun fibers mimics the natural extracellular matrix, creating an ideal microenvironment for cell growth and tissue regeneration [20,21]; (ii) through the selection of different polymer materials and parameters, the mechanical properties, degradation rates, and drug release kinetics can be precisely tailored to meet diverse clinical requirements [22]; and (iii) near-infrared (NIR) light-triggered photothermal effects exhibit superior spatiotemporal controllability, enabling precise localized treatment while minimizing damage to surrounding healthy tissues [13,14,23].

This review presents a systematic review of the emerging field of “photothermal electrospinning” and provides an in-depth analysis of material–property–function relationships, with particular emphasis on fiber fabrication methods, antibacterial applications, and non-antibacterial applications. The insights presented herein can serve as a valuable reference and inspiration for future research in the field of photothermal electrospinning.

## 2. Overview of Electrospinning

Spinning, a century-old textile manufacturing technology, can be classified into two main categories: electrospinning and alternative spinning techniques (commonly referred to as “non-electrospinning methods”) [24]. Electrospinning (ES) is an advanced fabrication method that utilizes high-voltage electric fields to create polymer solutions or to melt them into nanoscale to microscale fibers [25]. This technology can be traced back to the early observations of electrostatic phenomena and has evolved significantly since the late 20th century, with the advent of nanotechnology emerging as a pivotal tool in materials science [1,2]. Compared with other spinning techniques (e.g., solution blowing, centrifugal spinning, and drawing technique), the exceptional characteristics of electrospun fibers, including their high specific surface area, tunable porosity, controllable architecture, and excellent capacity for loading functional materials, have established their broad potential in biomedical applications [26,27,28,29,30]. In addition to well-documented conventional fiber fabrication methods, innovative strategies have emerged in recent years, attracting significant research attention. For instance, Makarov et al. [31] developed a composite fiber preparation approach combining dry-jet wet spinning with selective dissolution. Their method involved (i) co-dissolving polyacrylonitrile (PAN) and cellulose in N-methylmorpholine-N-oxide (NMMO) solvent to produce PAN/cellulose composite fibers via dry-jet wet spinning, followed by (ii) selective dissolution of the PAN matrix using dimethyl sulfoxide (DMSO) to obtain highly aligned cellulose microfibers (1–2 μm diameter). The resulting fibers exhibited superior mechanical properties (elastic modulus ~30 GPa) and Lyocell-like microstructures, making them suitable for composite reinforcement applications. While such innovative approaches demonstrate considerable academic merit, electrospinning remains the predominant fiber fabrication technique in biomaterials research due to its unparalleled advantages in fiber refinement, process simplicity, and scalability that better align with current practical requirements in the field.

**Figure 1:**
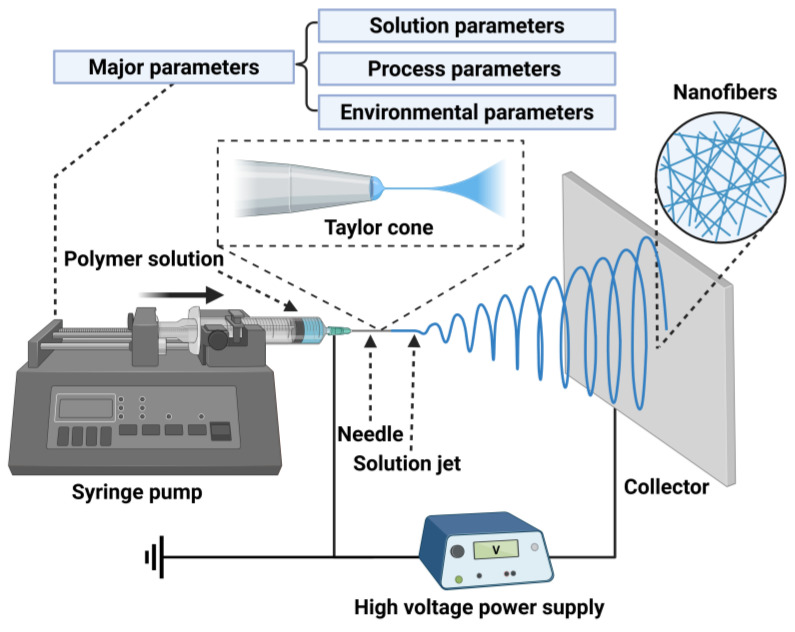
Schematic diagram of basic electrospinning apparatus, process and major parameters. Created in BioRender. Wang, K. (2025) https://BioRender.com/iuyyfek.

As shown in Figure 1, the electrospinning process represents a complex physicochemical phenomenon involving intricate interactions among electric field forces, surface tension, and fluid viscoelasticity. The process starts with the formation of charged polymer droplets at the spinneret, which progressively deform into characteristic Taylor cone structures under increasing electric field strength. When the electric force surpasses a critical threshold, charged jets eject from the cone apex, undergoing dramatic stretching and whipping instabilities during their trajectory toward the collector. This dynamic process results in rapid jet diameter reduction accompanied by solvent evaporation, ultimately yielding solidified ultrafine fibers that deposit onto the collector [25,32,33]. Various collector geometries (e.g., stationary flat plates, rotating cylinders, or mesh substrates) can be employed to obtain fibers with a specific alignment or to fabricate membranes with tailored 3D architectures [34]. Notably, fiber formation is governed by three primary parameter categories: (i) solution parameters (e.g., polymer concentration, molecular weight, conductivity, and surface tension), (ii) processing parameters (e.g., applied voltage, flow rate, and collecting distance), and (iii) environmental parameters (e.g., temperature and humidity). Complex interactions occur among these parameters, which have significant impacts on the final fiber morphology [33,35,36,37]. For example, an increase in polymer concentration correlates with larger fiber diameters and superior mechanical strength, which arises from intensified intermolecular interactions and physical chain entanglements [38,39]. Compared with nanoscale fibers, micro-scale fibers exhibit lower requirements for electrospinning conditions. However, an increased diameter reduces the specific surface area, consequently decreasing loading efficiency for functional materials. This process often involves complex trade-offs. Fiber diameter has been identified as the most critical characteristic, correlating with mechanical properties [40]. It is generally recognized that electrospun fibers exhibit exponential improvements in mechanical and other properties as their diameter decreases [41]. Yao et al. [42] fabricated poly(p-phenylene terephthalamide) (PPTA) fibers with diameters ranging from 275 nm to 15 μm using electrospinning. Their study revealed a significant increase in Young’s modulus and tensile strength with decreasing fiber diameter. The smallest fibers (2.1 μm) demonstrated notably enhanced mechanical properties, with tensile strength and Young’s modulus reaching 1.1 GPa and 59 GPa, respectively. Kim et al. [43] suggested that the mechanical improvement associated with reduced diameter may result from increased crystallinity in individual fibers and partial alignment along the winding direction, contributing to the high anisotropy in the mechanical strength of electrospun fiber mats. However, the relationship between fiber diameter and performance is not absolute. Yang et al. [44] found that in fibers with diameters below 200 nm, the orientation of “α”-phase crystals was disrupted due to rapid solidification during electrospinning. In contrast, fibers with a diameter of 300 nm exhibited the highest degree of “α”-phase crystal orientation, corresponding to the greatest mechanical strength and optimal mechanical performance.

Furthermore, maintaining all parameters within their optimal ranges is essential for successful fabrication [40]. Previous studies have demonstrated that Taylor cone instability, which may be caused by excessive concentration, viscosity, surface tension, or humidity, leads to bead formation. Alternatively, after the polymer fiber jet emerges from the Taylor cone, non-uniform deposition along the jet may occur, creating polymer-rich and polymer-deficient regions that ultimately develop into bead-on-string structures [45]. Highly beaded fibers are generally considered defective as they adversely affect mechanical properties [46]. Numerous studies have been conducted to overcome the generation of beads-on-the-string by optimizing experimental conditions. However, recent studies have revealed the unique functional advantages of beaded fibers, demonstrating that drug release profiles can be modulated by precisely controlling bead dimensions. As exemplified by Li et al. [47], a Janus beaded fiber system was developed using polyvinylpyrrolidone K90 (PVP K90) and ethylcellulose (EC) as polymer matrices to co-load ketoprofen (KET) and methylene blue (MB) as model drugs. In vitro dissolution tests showed that this Janus architecture enabled dual-drug controlled release through distinct mechanisms: erosion-based release from the linear MB-PVP component and Fickian diffusion-dominated release from the beaded KET-EC compartment.

**Figure 2:**
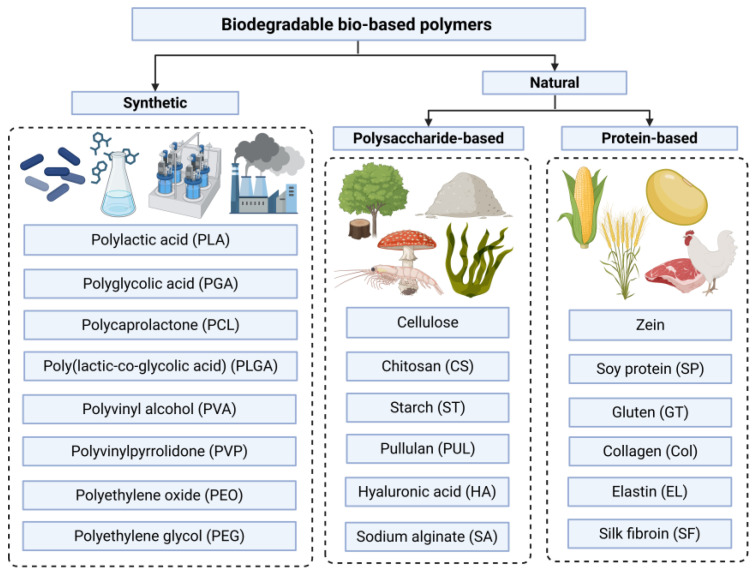
Two main categories of biodegradable bio-based polymers for electrospun fibers. Created in BioRender. Wang, K. (2025) https://BioRender.com/39idxtt.

The post-World War II era witnessed the emergence of petroleum-based polymers as a driving force for global economic development, including polyvinylidene fluoride (PVDF), polypropylene (PP), polysulfone (PSF), and polyether sulfone (PES). However, the inherent toxicity and non-biodegradability of these polymers has led to significant challenges in environmental and biomedical applications. An increasing amount of research efforts have been directed toward developing environmentally friendly, biodegradable, and readily available alternatives [48,49]. Currently, polymers suitable for producing electrospun fibers for biomedical applications can be divided into two categories: synthetic polymers and natural polymers (Figure 2) [37,50]. Synthetic polymers are not directly extracted from biological sources but are synthesized through fermentation processes or chemical reactions utilizing renewable and biodegradable raw materials [51]. Among synthetic polymers, aliphatic polyesters such as polylactic acid (PLA), polyglycolic acid (PGA), polycaprolactone (PCL), and poly(lactic-co-glycolic acid) (PLGA) have garnered the most extensive research and applications [52]. In addition, polyvinyl alcohol (PVA) [53], polyvinylpyrrolidone (PVP) [54], polyethylene oxide (PEO) [55], and polyethylene glycol (PEG) [56] are commonly used synthetic polymers for electrospinning. Unlike synthetic polymers, natural polymers are directly sourced from biological materials and can be extracted and processed using biotechnological approaches [51]. These polymers are broadly classified into two main categories [37,57]: (i) polysaccharide-based natural polymers, including cellulose [58], chitosan (CS) [59], starch (ST) [60], pullulan (PUL) [61], hyaluronic acid (HA) [62], and sodium alginate (SA) [63], and (ii) protein-based natural polymers, including zein [64], soy protein (SP) [65], gluten (GT) [66], collagen (Col) [67], elastin (EL) [68], and silk fibroin (SF) [69].

Although both synthetic and natural polymers are biomass-derived, their divergent material characteristics and associated drawbacks substantially limit practical applications. Synthetic polymers offer substantial flexibility and controllability in synthesis and modification, yet they frequently suffer from inadequate hydrophilicity and a lack of cell surface recognition sites, resulting in poor cellular affinity. In contrast, natural polymers demonstrate superior biocompatibility and low immunogenicity, with some even exhibiting intrinsic antimicrobial properties. However, their applications are limited by poor processability, insufficient mechanical strength, and high solubility [37]. To overcome these limitations, researchers have developed advanced strategies such as blending with biomacromolecules, compositing with inorganic materials, and crosslinking with functional components. Notably, hybrid systems that combine synthetic and natural polymers have emerged as a particular promise, offering an optimal balance between mechanical robustness and preserved bioactivity [4,50,70,71,72].

Beyond intrinsic polymer properties, material selection requires comprehensive consideration of two primary factors: processing parameters and functional agent characteristics. Although natural polymers exhibit superior biocompatibility, they demand more stringent processing conditions than synthetic counterparts. Studies reveal that electrospun nanofibers of natural extracellular matrix (ECM) components (e.g., pure collagen) lose their native fibrous architecture. This detrimental outcome stems not only from organic solvent effects but also from potential biochemical degradation during electrospinning [73]. Furthermore, functional groups in natural polymers often complicate electrospinning. SA demonstrates rigid chain conformation, high aqueous viscosity, and insufficient chain entanglement, collectively impeding fiber formation [74]. CS exhibits poor solubility in common solvents, while strong intermolecular/intramolecular hydrogen bonding elevates solution viscosity, hindering the ability to overcome surface tension during electrospinning [75]. Polymatrix–functional agent compatibility (exemplified here by PTAs) represents another critical consideration for developing stable, homogeneous electrospun systems. For instance, crystalline polymers such as PCL and PLA are well suited for incorporating inorganic PTAs (e.g., Au and MXene) due to their high mechanical strength, which prevents nanoparticle agglomeration, and their hydrophobic surfaces, which minimize PTA leakage. However, excessive PTA loading may compromise fiber integrity, leading to breakage.

## 3. Overview of Photothermal Agents (PTAs) and Photothermal Therapy (PTT)

**Figure 3:**
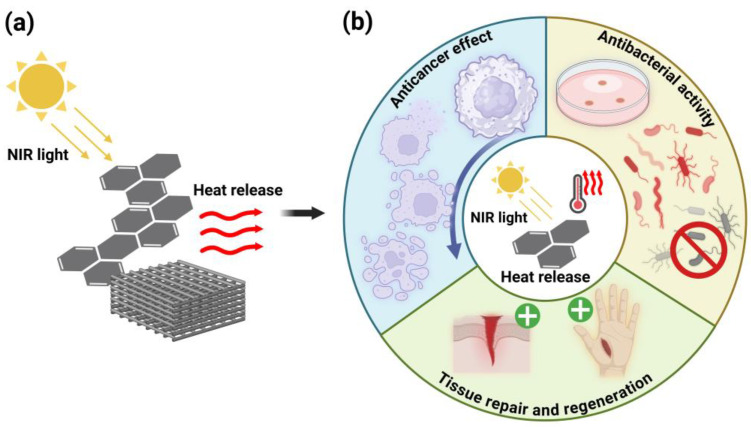
Mechanism and biomedical applications of photothermal therapy (PTT). (**a**) Under NIR light irradiation, photothermal agents (PTAs) convert light energy into heat energy and release it, resulting in localized high temperatures; (**b**) PTT’s biomedical applications mainly include bacterial eradication, cancer treatment, and tissue regeneration. Created in BioRender. Wang, K. (2025) https://BioRender.com/orkpy29.

Photothermal therapy (PTT) is a highly promising therapeutic technique that utilizes photothermal agents (PTAs) to convert near-infrared (NIR) light energy into heat, generating localized hyperthermia [12]. In the biomedical field, PTT has attracted growing research interest due to its remarkable potential in bacterial eradication, cancer treatment, and tissue regeneration applications (Figure 3).

The radiation employed in PTT typically falls within the NIR range (650–1024 nm), with 808 nm being the most frequently used wavelength [13,76]. Compared with ultraviolet–visible (UV–Vis) light, biological tissues such as skin and hemoglobin exhibit minimal absorption and scattering of NIR light, as NIR wavelengths lie within the primary region of the so-called “biological transparency window”. This characteristic enables NIR light to achieve deeper tissue penetration (up to 1 cm) without causing adverse effects on tissues and organs, making it particularly attractive for biomedical applications [14,23].

**Figure 4:**
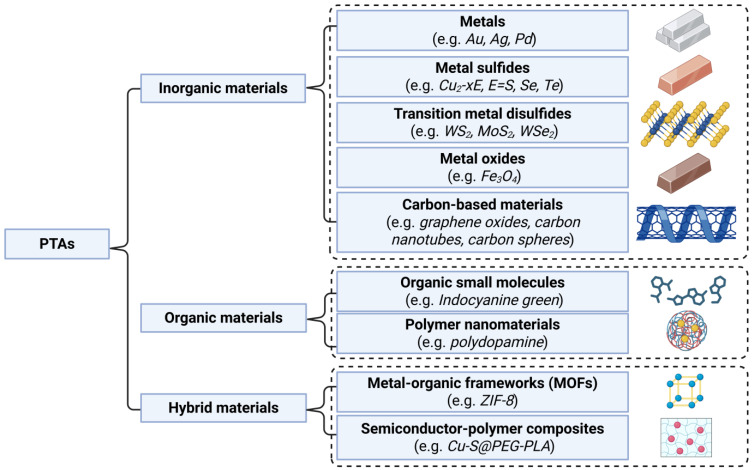
Different classifications of photothermal agents (PTAs) used for PTT. Created in BioRender. Wang, K. (2025) https://BioRender.com/x3a49kc.

PTAs serve as the core components enabling PTT functionality. Based on their composition, PTAs can be classified into inorganic, organic, and hybrid materials (Figure 4). Inorganic materials represent the most commonly used category of PTAs, including metals (e.g., Au, Ag, and Pd), metal chalcogenides (e.g., Cu_2−x_E, E = S, Se, and Te), transition metal dichalcogenides (e.g., WS_2_, MoS_2_, and WSe_2_), metal oxides (e.g., Fe_3_O_4_), and carbon-based materials (e.g., graphene oxide, carbon nanotube, and carbon sphere). Among organic materials, small molecules such as indocyanine green and polymeric nanomaterials such as polydopamine have shown particular promise. Hybrid materials, including metal–organic frameworks (MOFs) and semiconductor–polymer composites (e.g., Cu-S@PEG-PLA), have received increasing attention in recent years [15,77,78].

The majority of PTAs convert light energy into heat through non-radiative relaxation processes. The fundamental mechanism involves PTAs absorbing photons of specific wavelengths, followed by excited-state electrons releasing energy as heat through lattice vibrations or intramolecular vibrational relaxation. However, in noble metals and certain narrow-bandgap semiconductors, the generation of surface plasmon resonance (SPR) effects enhances local electromagnetic fields, enabling energy conversion to heat through electron–electron scattering and electron–phonon interactions. For organic small molecules, energy dissipation occurs through molecular vibrational relaxation, where photoexcited electrons undergo transitions, followed by internal conversion (IC), to transform energy into vibrational levels, ultimately releasing it as heat. Additionally, in some semiconductor materials, photo-generated electron–hole pairs captured by defect states or surface states can release thermal energy through non-radiative recombination [79,80].

The cytotoxic effects of hyperthermia on cells can be exemplified by the fundamental process of gold nanoparticle (AuNP)-mediated PTT for cancer treatment. The unique SPR effect of AuNPs enables all cancer cells to be exposed to localized high temperatures under light irradiation, giving PTT significant potential in cancer therapy. The size of AuNPs represents the most critical factor influencing nanoparticle performance, affecting both cellular uptake efficiency and photothermal conversion characteristics [81]. For instance, small AuNPs (up to 15 nm) are readily absorbed by intestinal epithelial cells. Studies on the size dependence of SPR in gold nanospheres (20–100 nm range) have shown that absorption efficiency increases with NP size, with 70 nm particles demonstrating optimal efficiency [82].

AuNPs can accumulate in tumor tissues through passive mechanisms such as the enhanced permeability and retention (EPR) effect, or through active targeting by conjugating with chemical moieties (e.g., monoclonal antibodies or folic acid) that are overexpressed in cancer cells [16,83,84]. AuNP-mediated PTT involves multiple mechanisms, primarily the induction of cancer cell necrosis (through structural changes in proteins and lipids) and apoptosis (via increased gene expression) [85]. When heated to temperatures between 41 and 47 °C, cells undergo apoptosis within one hour, while excessive cell necrosis caused by thermal shock typically above 50 °C leads to much faster cell death (on the order of minutes), primarily through protein denaturation [84,86].

Beyond specific tumor ablation, the photothermal process importantly enhances immune responses following the highly immunogenic thermal death of cancer cells, contributing to long-term anti-tumor efficacy [87]. Existing research has demonstrated that combining hyperthermia with radiotherapy or chemotherapy can improve treatment outcomes. In the treatment of metastatic head and neck squamous cell carcinoma, concurrent radical radiotherapy and hyperthermia significantly enhanced therapeutic efficacy without noticeably increasing the toxic side effects [88,89]. Similarly, combination regimens of hyperthermia and chemotherapy have shown comparable sensitization effects. Particularly noteworthy is that specific therapeutic agents for malignant melanoma, when combined with hyperthermia at different temperature conditions (43 °C and 45 °C), can effectively activate endogenous or exogenous ER pathways, thereby promoting tumor cell apoptosis [90].

## 4. Fabrication Methods of Photothermal Electrospun Fibers

Photothermal electrospinning (PTE) is an advanced technique that combines electrospinning (ES) with photothermal therapy (PTT). In this method, photothermal agents (PTAs) are embedded within electrospun fibers to generate targeted heat when exposed to near-infrared (NIR) irradiation. Compared with conventional injectable photothermal nanoparticles, electrospun fiber-based patches or dressings can physically adhere to target tissues, prolonging the localized retention of PTAs and reducing systemic toxicity caused by blood circulation [91]. The electrospinning technique also enables co-loading of PTAs with antibiotics or chemotherapeutics for synergistic therapy. Although hydrogels can similarly deliver PTAs, electrospun fibers with tunable stiffness (0.1–100 MPa) avoid mechanical mismatch in load-bearing applications, offering superior biomimetic properties [92].

**Figure 5:**
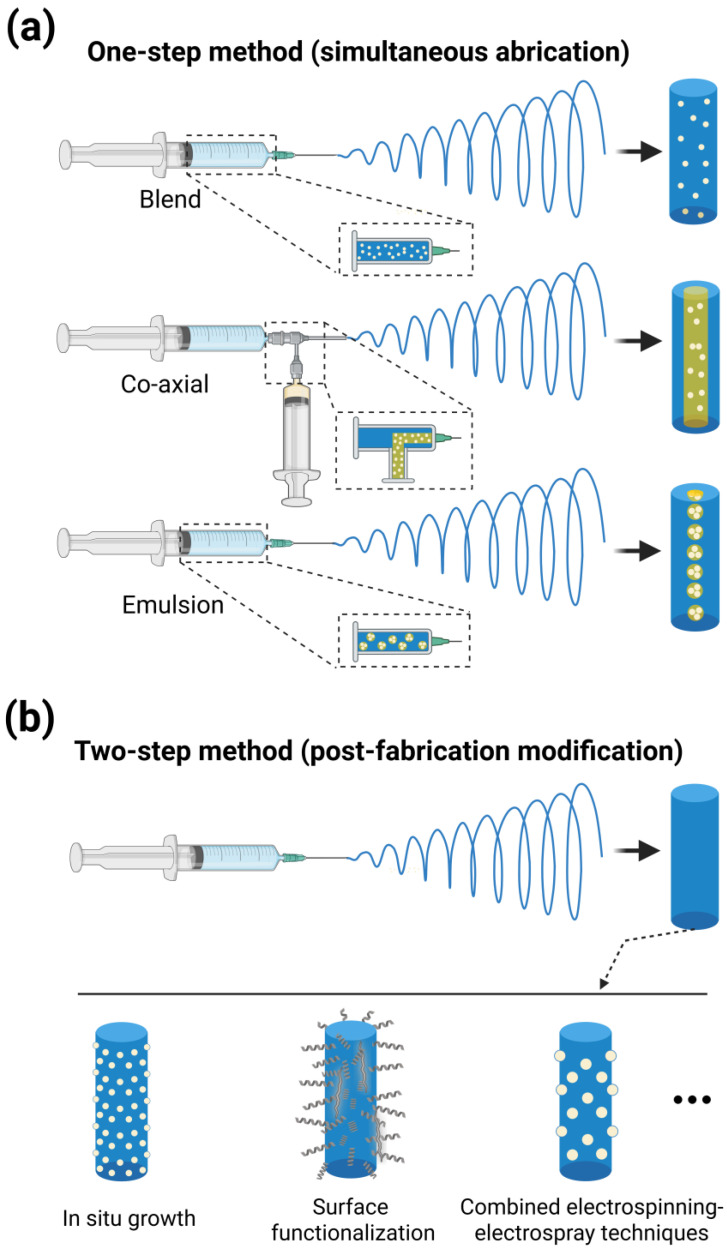
The two main methods for incorporating PTAs into electrospun fibers. (**a**) One-step methods primarily involving blend electrospinning, co-axial electrospinning, and emulsion electrospinning techniques; (**b**) Two-step methods such as in situ growth, surface functionalization, and combined electrospinning-electrospray techniques. Created in BioRender. Wang, K. (2025) https://BioRender.com/k7sglkz.

Two principal methodologies exist for incorporating photothermal agents (PTAs) into electrospun fibers (Figure 5): (i) the one-step method (simultaneous fabrication), where polymers and PTAs or other functional components are directly co-electrospun using techniques such as blend, co-axial, or emulsion electrospinning, and (ii) the two-step method (post-fabrication modification), which involves initial fabrication of the fiber matrix, followed by surface modification with PTAs or other functional ingredients through some chemical immobilization or physical modification approaches [93,94,95,96].

### 4.1. One-Step Method

Blend electrospinning has emerged as the most prevalent technique for fabricating photothermal electrospun fibers, owing to its simplicity in requiring only the dissolution of both fiber-forming polymers and PTAs within a common solvent system using conventional electrospinning apparatus. The compatibility and interfacial tension between polymers and PTAs critically determine the homogeneity of PTA dispersion in the solution system, thereby influencing fiber morphology and functionality. Although blending is the simplest and most common preparation method, uneven distribution and burst release of PTAs and bioactive molecules in fibers limit their practical efficacy [97]. Recent research trends have focused on incorporating multiple functional components into electrospun fibers to achieve enhanced therapeutic outcomes. While simple blending of PTAs enables photothermal conversion for pathogen or cancer cell ablation, the complexity of pathological microenvironments often necessitates combination with additional therapeutic agents (e.g., antibiotics [98] and chemotherapeutics [99]) or multifunctional PTA-based composites (e.g., hybrid nanoparticles [100,101,102,103,104]) to achieve complete therapeutic efficacy. A representative study demonstrated this approach through the development of a “triad” fiber for infected bone defect treatment, incorporating photothermal agents, silver nanoparticles (AgNPs), and dexamethasone (DEX) into poly(L-lactic acid) (PLLA) via blend electrospinning. The AgNPs functioned dually as both antibacterial agents (through direct contact or Ag^+^ release) and photothermal converters (generating localized hyperthermia and singlet oxygen (^1^O_2_) under 808 nm irradiation). DEX, a well-established osteoinductive drug, promoted mesenchymal stem cell (MSC) differentiation into osteoblasts, thereby accelerating bone defect healing. The PLLA electrospinning solution exhibited high viscosity and surface tension, preventing sufficient jet elongation under electric forces, resulting in fibers with an average diameter of 219 ± 15 nm. Upon incorporating Ag-NPs or Dex, the solution conductivity increased, leading to significantly reduced fiber diameters. Notably, the PLLA/Dex/Ag fibers displayed a uniform morphology with smooth surfaces, no adhesions, and the smallest diameter (162 ± 6 nm). Their high specific surface area contributed to enhanced hydrophilicity [105].

Core–shell fiber fabrication primarily utilizes two established electrospinning techniques with different formation mechanisms, which requires a more complex process in most cases [97,106]. The co-axial method employs concentric needles to simultaneously extrude distinct solutions under high voltage, directly yielding core–shell structures through controlled interfacial tension [107]. Research indicates that successful coaxial electrospinning critically depends on maintaining a sharp and well-defined core–shell interface with a constant, minimized interfacial energy at the boundary. Ideally, the core and shell materials should exhibit partial miscibility [108]. Additionally, the flow rate ratio between the two phases must be precisely controlled to prevent fiber breakage or the formation of defective fibers. Emulsion electrospinning achieves similar morphologies by electrospinning homogeneous blends of immiscible liquids, where the dispersed phase’s volatility governs the final fiber architecture [109]. In contrast, emulsion systems exhibit significantly poorer stability, often requiring immediate electrospinning or stabilizers to prevent phase separation and impaired fiber formation [97]. Nevertheless, emulsion electrospinning remains a promising alternative for core–shell fiber production due to its compatibility with both hydrophilic and hydrophobic polymers, along with its ability to function without specialized coaxial spinnerets [106].

The strategic loading of therapeutic agents in the core and PTAs in the shell enables controlled drug release. The underlying mechanism involves temperature-induced expansion of the free volume through enhanced polymer chain mobility when exceeding the shell material’s glass transition temperature (T_g_). Azerbaijan et al. [110] demonstrated this concept using poly(tetramethylene ether) glycol-based polyurethane (PTMG-PU) core–chitosan shell nanofibers co-loaded with paclitaxel (PTX) and graphene oxide/gold nanorods (GO/Au NRs). Their study revealed that 10 wt.% PTMG-PU and 4 wt.% chitosan represented the optimal core–shell solution concentrations, producing fibers with a diameter of 180 nm. Deviations from these concentrations resulted in either larger fiber diameters (at higher concentrations) or electrospinning difficulties (at lower concentrations). The chitosan shell prevented an initial burst release, while acidic pH (through weakened carboxyl groups and matrix swelling) and NIR irradiation (through photothermally enhanced chain mobility) synergistically accelerated PTX release. Moreover, Park et al. [111] developed polycaprolactone (PCL) fibers with Au nanocage-loaded shells containing fatty acid eutectic mixtures with a melting point of 39 °C, where NIR-induced heating melted the PCM to enable rapid drug release. The produced fibers exhibited smooth surfaces with homogeneous drug distribution and an average diameter of 2.84 ± 0.12 μm. A hollow core structure was formed within the fibers, serving as a reservoir for hydrophilic drugs. This unique morphology resulted from the significant surface energy difference between the aqueous droplets and chloroform phase in the W/O emulsion system.

### 4.2. Two-Step Method

The two-step method offers advantages for incorporating components sensitive to high voltage or organic solvents, preserving additive functionality while maintaining the polymer matrix’s original degradation rate and mechanical properties [94,95].

In situ growth techniques enable direct nanoparticle formation on/within fiber matrices through chemical reactions or physical deposition. For instance, fiber scaffolds immersed in dopamine solution allow for molecular infiltration, followed by Tris-HCl buffer (pH 8.5)-initiated polymerization to form polydopamine nanoparticles uniformly coating fiber surfaces with photothermal functionality [112,113,114]. Metal–organic frameworks (MOFs) such as ZIF-8 are particularly suitable due to their thermal stability. The pH-responsive dissolution of ZIF-8’s imidazole ligands under acidic conditions (e.g., tumor or infected microenvironments) enables controlled drug delivery [115,116,117]. Beyond external protective coatings, ZIF-8’s Zn^2+^-methylimidazole coordination structure provides high porosity and controlled release capacity, exemplified by its successful encapsulation of FDA-approved indocyanine green (ICG) through sulfonic acid–zinc ion coordination [118]. Although in situ growth offers a facile surface modification approach, nanoparticle growth within fibers often leads to excessive aggregation, resulting in uncontrolled particle sizes [119].

Surface functionalization represents another common post-electrospinning modification strategy. Plasma technology represents a dry, eco-friendly, and worker-safe approach for surface modification without altering the bulk properties of materials. Plasma treatment using oxygen, nitrogen, or air plasma modifies fiber surface physics and chemistry through high-energy particle interactions. The introduced polar functional groups and increased roughness enhance surface energy, wettability, and hydrophilicity, all of which promote cell adhesion and tissue regeneration. Notably, these polar groups enable the covalent conjugation of functional materials [120,121]. Mauro et al. [122] employed N_2_ plasma to introduce amine groups on PCL fibers for covalent graphene oxide (GO) deposition via reactions with inherent epoxy groups. After N_2_ plasma treatment, the microfibers showed no significant structural damage but exhibited surfaces characterized by rough and porous regions. The GO sheets adhered tightly to the microfiber surfaces, even bridging adjacent microfibers. The GO coating provided both photothermal ablation of tumors and reduced metastatic potential through enhanced cancer cell adhesion to the fibers. Despite its significant potential in biomedical applications, plasma technology still faces two critical limitations: (i) gradual performance degradation due to material aging post-treatment and (ii) the lack of compatible equipment for scalable processing [123]. Alternative approaches include direct incorporation of the linker components for functional agents. In a study, blended polyethyleneimine (PEI)’s abundant amines enabled the Michael addition of sulfobetaine methacrylate (SBMA) to create zwitterionic surfaces that effectively resisted protein adsorption and biofilm formation in wound dressings. The membrane containing 10 wt.% PEI exhibited a uniform structure with fiber diameters ranging from 310 to 590 nm. These fibers demonstrated high surface porosity (>90%) and pore sizes averaging 500–900 nm, closely mimicking the dimensions of natural collagen fibers and skin tissue [124].

Electrospraying is a versatile technique that utilizes electrical forces to atomize liquids into fine droplets. This method offers simplicity, precise controllability, and mild processing conditions, enabling the integration of functional materials as microparticles within or on fiber scaffolds through combined electrospinning–electrospray techniques [125,126,127]. Zhang et al. [128] demonstrated spontaneous embedding of CNT@ZIF composites into PLA fibers at 30 kV with 1 mL/h (PLA) and 0.75 mL/h (CNT@ZIF) feed rates. Alternatively, coaxial electrospraying created vancomycin-loaded PCM (lauric acid–stearic acid = 8:2) microparticle antimicrobial coatings on Apt-PCL/BP fiber scaffolds, where NIR-induced heating triggered PCM solid–liquid transition for rapid antibiotic release. The formation of protruding CNT@ZIF-8 nanohybrids embedded in fiber surfaces created significant surface roughness. Increasing the CNT@ZIF-8 mass fraction elevated the electrospinning solution’s surface tension, resulting in larger fiber diameters while maintaining nanoscale dimensions [102]. Similar to electrospinning, electrospraying involves electrical stresses and requires volatile organic solvents, both of which may compromise the structural integrity and functionality of sensitive biomolecules (e.g., proteins, enzymes, and DNA plasmids). Consequently, comprehensive safety evaluations of all electrospraying parameters are warranted [126]. Additional surface functionalization methods, including solution casting [111], dip coating [129], and self-assembly [130], have been reported but warrant less detailed discussion here.

## 5. Biomedical Applications of Photothermal Electrospun Fibers

### 5.1. Antibacterial Applications

The escalating prevalence of antibiotic resistance has been recognized as one of the top ten “global public health threats” by the World Health Organization. Prolonged and repetitive systemic antibiotic administration represents the primary driver of multidrug-resistant (MDR) bacterial emergence, leading to the failure of conventional therapeutic approaches. Furthermore, the systemic toxicity and adverse effects associated with excessive antibiotic usage cannot be overlooked. These pressing concerns have necessitated the development of novel antibacterial strategies [14,131].

**Figure 6:**
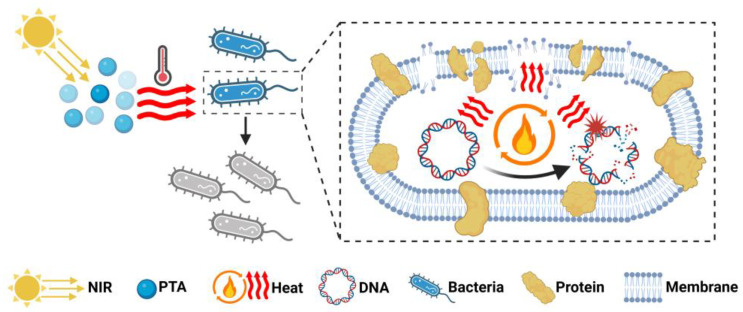
Schematic illustration of the mechanism of antibacterial PTT. Created in BioRender. Wang, K. (2025) https://BioRender.com/6y344a8.

In recent years, photothermal therapy (PTT) has demonstrated considerable potential in combating bacterial infections (Table 1) [132]. As the fundamental component of PTT, photothermal agents (PTAs) convert light energy into thermal energy, inducing membrane disruption, protein denaturation, and irreversible bacterial destruction (Figure 6) [131,133].

The simplest PTT implementation involves blending PTA-based materials into electrospun fibrous membranes as wound dressings, which upon NIR laser irradiation, generate localized hyperthermia. Tian et al. [100] developed Au@CD composite nanoparticles (comprising gold nanoparticles (AuNPs) and N,S-doped carbon dots (CDs)) incorporated into PVA electrospun membranes. Compared with pristine AuNPs, the CD modification enhanced photothermal conversion efficiency, photostability, and biocompatibility. Under NIR irradiation, local temperatures reached 50 °C, achieving >99% inactivation rates against both S. aureus and E. coli, along with superior wound-healing outcomes. Fu et al. [101] encapsulated MXene within GelMA microgels to improve wound-site stability, subsequently blending them with chitosan/gelatin polymers to fabricate microgel/nanofiber membranes via electrospinning. These dressings enabled programmable PTT and mild photothermal therapy (MPTT) under rapid sterilization (5 min at 52 °C) and mature biofilm dispersion (10 min), coupled with stimulated fibroblast proliferation and enhanced angiogenesis through controlled mild heating (42 °C). Cai et al. [134] employed silk fibroin as a biotemplate to synthesize silk fibroin–copper sulfide nanoparticles (SF/CuS NPs), which were incorporated into PVA nanofiber membranes. This composite retained CuS NPs’ excellent photothermal responsiveness while gaining improved hydrophilicity, biocompatibility, and wound-healing properties from silk fibroin. Chen et al. [133] designed a multifunctional PLGA electrospun membrane incorporating MoS_2_@Pd for diabetic wound management, combining anti-inflammatory, antioxidant, and antibacterial properties. The MoS_2_ component provided efficient NIR photothermal conversion and ROS scavenging through intrinsic enzyme-mimicking activity, while Pd doping enhanced catalytic performance. This membrane demonstrated 97.14% and 97.07% inhibition rates against S. aureus and E. coli, respectively, along with suppressed bacterial growth, reduced inflammation, and optimal wound healing in diabetic rat models.

However, achieving complete sterilization through PTT alone often requires prolonged high-temperature exposure that may damage surrounding healthy tissues. Combining PTT with other modalities—such as photodynamic therapy (PDT), chemotherapy, and nitric oxide (NO)-based gas therapy—has emerged as a promising solution. This synergistic approach enhances antibacterial efficacy within tissue-tolerable temperature ranges while reducing PTA dosage and heating duration, creating complementary therapeutic effects wherein “1 + 1 > 2” [14,135].

Recent studies have demonstrated superior bactericidal efficiency when combining PTT with PDT compared with PTT alone, which is considered the most promising combined therapy. Unlike PTT, PDT primarily relies on reactive oxygen species (ROS) generated by photosensitizers (PSs) to damage bacterial lipids, proteins, and DNA. Elevated temperatures may increase bacterial permeability, facilitating PS uptake and enhancing PDT efficacy. Conversely, PS-generated ROS can reduce bacterial thermotolerance, amplifying PTT effects [135,136]. However, separate administration of PTAs and PSs cannot achieve synergistic effects in vivo due to differing light source requirements [137]. The molecular-level design of PTT–PDT coupled materials has therefore become crucial, enabling single NIR light source activation.

Zhang et al. [138] developed MXene/ZIF-8 nanoassemblies dispersed in polylactic acid (PLA) electrospun membranes. The MXene/ZIF-8 heterostructure facilitates intermolecular charge transfer, improving intersystem crossing rates and stabilizing excited states for enhanced NIR sensitization, thereby simultaneously generating hyperthermia and ROS for antibacterial and tissue-regenerative effects. Another study created a coupled material by electrostatically adsorbing positively charged IR780 PS onto negatively charged MoS_2_ nanosheets, which were then blended into PVA nanofibers. This system exhibited broad-spectrum antibacterial activity at relatively low temperatures (52.7 °C for 90 s) through dual PTT–PDT mechanisms [139]. Similarly, Sun et al. [137] fabricated upconversion nanoparticle@TiO_2_@GO (UCNPs@TiO_2_@GO) composites that were electrospun with polyvinylidene fluoride (PVDF) to create nanocomposite membranes capable of concurrent temperature elevation and ROS generation under single-wavelength NIR irradiation.

Beyond PDT, combining PTT with chemotherapy has shown potential for synergistic antibacterial treatment. PTT-induced heat can trigger thermo/NIR-responsive drug release while enhancing bacterial drug uptake and reducing resistance. Conversely, chemotherapeutic agents may lower bacterial heat resistance [135]. PTT–chemotherapy is less commonly used in antibacterial applications due to the mismatch between the systemic toxicity of antibiotics and the precision of local photothermal therapy. Federico et al. [98] developed hyaluronic acid derivative (HA-EDA) electrospun membranes loaded with graphene oxide (GO) and ciprofloxacin (CPX). NIR irradiation activated both photothermal effects and light-triggered CPX release, effectively reducing S. aureus and P. aeruginosa counts while disrupting preformed biofilms. Metallic nanoparticles (NPs) and their released ions can also synergize with photothermally generated heat. Among various metal-based nanomaterials, Ag-based systems have been most widely applied as antibiotic alternatives against MDR pathogens. Zhao et al. [140] fabricated polycaprolactone (PCL) nanofibers incorporating AgNPs and black phosphorus (BP). The AgNPs mediated bacterial membrane permeability and ROS generation, while BP-derived hyperthermia not only directly killed bacteria but also accelerated Ag^+^ release kinetics—together enhancing antibacterial efficiency. Another study embedded silver nanowires (AgNWs) into PLA nanofibers, where NIR-triggered Ag^+^ release and localized photothermal effects conferred excellent antibacterial properties [141]. Tang et al. [142] incorporated lanthanum chloride (LaCl_3_) into PVA/CS/GO nanofibers, improving hydrophilicity, tensile strength, and antibacterial performance (82% and 99.7% inhibition against S. aureus and E. coli, respectively), with further enhancement after 20 min of 808 nm NIR irradiation. Qiu et al. [143] adopted a different approach by self-assembling PDA–Zn–Ag coatings onto PLLA/HANW composite membranes, achieving nearly 100% inhibition against both E. coli and S. aureus through combined NIR-induced PTT and synergistic Zn^2+^/Ag^+^ release.

Nitric oxide (NO), a gaseous molecule with broad-spectrum bactericidal activity, has shown potential for antibacterial therapy. NO directly modifies membrane proteins and induces DNA damage/protein dysfunction through oxidative byproducts such as N_2_O and ONOO^−^. Despite its wound-healing potential, clinical NO application faces challenges, including short half-life, high volatility, and inflammatory burst release. Consequently, exogenous stimulus-controlled NO release systems have attracted growing interest [144,145,146]. NIR-triggered NO release is particularly appealing due to its deep tissue penetration and minimal invasiveness, allowing for synergistic antibacterial action with concurrent PTT. Li et al. [124] integrated NO donor BNN6 into mesoporous polydopamine (MPDA) and electrospun it with PCL/PEI nanofibers. Under 808 nm irradiation, photothermally triggered NO release effectively targeted and disrupted biofilms. Similarly, Wang et al. [134] incorporated sodium nitroprusside-doped Prussian blue nanoparticles and type I collagen into CS/PVA nanofibers, where NIR-triggered NO release and photothermal conversion exhibited synergistic antibacterial activity alongside collagen-mediated wound repair. Beyond high-concentration antibacterial applications, low-concentration NO exhibits multiple functions, including vasodilation, angiogenesis promotion, signaling, and immunomodulation—all beneficial for wound healing [147,148]. Su et al. [149] leveraged this concentration-dependent duality by electrospinning PLLA fibers containing NO-loaded HKUST-1 particles. As an NIR-responsive MOF, HKUST-1 stored NO as NONOates formed with secondary amines on its modified surface, which degraded above 40 °C. During initial infection control, NIR-triggered massive NO release synergized with PTT to eliminate biofilms, while subsequent gradual NO release (during fiber degradation) cooperated with copper ions from MOF decomposition to promote healing.

Beyond these mainstream strategies, several innovative antibacterial approaches have been reported in photothermal electrospinning. Lai et al. [150] designed a self-activating composite dressing combining piezoelectric (PLLA) and photothermal (rGO) layers. Beyond NIR-triggered photothermal effects, ultrasound irradiation induced controlled ROS generation for antibacterial activity, while a CaO- and catalase-incorporated enzyme converted ROS into O_2_, alleviating chronic hypoxia in infected wounds. Certain materials also exert physical antibacterial actions through direct contact. For instance, BP nanosheets demonstrated both efficient photothermal conversion and direct bacterial killing via “nano-knife” effects. Additionally, positively charged quaternized chitosan (QCS) electrostatically disrupted negatively charged bacterial membranes through inherent antimicrobial properties [130].

**Table 1:** Photothermal electrospun fibers for antibacterial application.

Polymers	Photothermal Agents	Other Additives	Temperature Reached	Application Field	Ref.
PVA	Au@CD	-	About 50 °C	Wound healing	[100]
CS/GA	GelMA@MXene	-	PTT: 50–52 °C MPTT: 40–42 °C	Wound healing	[101]
PLA	MXene/ZIF-8	-	Above 45 °C within 25 s	Wound healing Tumor therapy	[138]
PVA/CS/HTCC	polyaniline (PANI)	S-Nitrosoglutathione (GSNO)	Approximately 58 °C within 30 s A stable plateau at 62 °C	Wound healing	[151]
PVA	MoS_2_-LA-COS	-	60.5 °C	Personal protective equipment	[152]
PVA	MoS_2_-IR780	-	52.7 °C within 90 s	Antibacterial therapy	[139]
PP (inner layer) PAN (outer layer)	PDA	-	Outer layer: 60 °C Inner layer: 30 °C	Wound healing	[153]
PCL (inner layer) PCL (outer layer)	TA-Fe^3+^	-	40−50 °C within 10 min	Wound healing	[154]
PCL	TA-Fe^3+^	-	41–45 °C within 10 min	Wound healing	[155]
PCL	BP	AgNPs	41 °C	Wound healing	[140]
PCL/Gel	ZIF-8	Ciprofloxacin hydrochloride (CIP)	47.8 °C	Wound healing	[156]
PCL	Mesoporous polydopamine (MPDA)	Sulfobetaine (SBMA) Polyethyleneimine 10,000 (PEI)	Increased 21.2 °C compared to original	Wound healing	[124]
PVDF	UCNPs@TiO_2_@GO	-	59.7 °C within 5 min	Wound healing	[137]
CS/PVA	SNP-PB NPs	Type I collagen	About 75 °C	Wound healing	[157]
PVA	SF/CuS NPs	-	51.5 °C	Wound healing	[134]
PLGA	MoS_2_@Pd	-	57 °C	Wound healing	[133]
PVA/PEO/CT/Chitin	Demineralized mussel shells (deMS)	-	70–95 °C	Contact lenses	[158]
Poly(3-hydroxybutyrate-co-3hydroxyvalerate) (PHBV)	Indocyanine green (ICG)	-	65 °C	Face Masks	[159]
PLLA	Ag NPs	Dexamethasone (Dex)	51.9 °C	Bone infection treatment	[105]
Hyaluronic acid derivative (HA-EDA)	GO	Ciprofloxacin (CPX)	47 °C	Wound healing	[98]
PLLA	NO@HKUST-1	-	42 °C	Wound healing	[149]
PLLA/QCS	BP	Hemoglobin (Hb)	40.1 ± 0.3 °C within 3 min	Wound healing	[130]
PVDF/HFP	AIEgen TTVB	-	-	Personal protective equipment (PPE)	[136]
PCL	AuNPs	-	-	Wound healing	[160]
2-MI/PLA	Cur-ICG@ZIF-8	-	46 °C	Wound healing	[118]
PLA	CNT@ZIF-8	-	45.7 °C within 100 s	Personal protective equipment	[128]
PCL	uCNT@PDA	Cur	52–115 °C (uCNT@PDA: 0.25% w/v-1.0% w/v)	Bacterial eradication	[161]
PAN	AuNPs	-	60 °C	Personal protective equipment Surgical face masks	[162]
PLA	AgNWs	-	About 40 °C	Wound healing	[141]
Thermoplastic polyurethane (TPU)	EGaIn(liquid metal/Gallium (Ga)/Indium (In)/Tin (Sn))	Cur-loaded LA nanoparticles	About 45 °C	Wound healing	[163]
PLLA	PDA-Zn-Ag bimetallic coating	Hydroxyapatite nanowires (HANWs)	53.5 °C	Wound healing	[143]
PLLA	Reduced graphene oxide (rGO)	Calcium peroxide (CaO_2_) and catalase (CAT)	About 50–55 °C	Wound healing	[150]
PAN/PEG	Cu_2_O/V_2_CTx	-	60 °C	Wound healing	[164]
PVA/CS	GO	LaCl_3_	Over 160 °C within 15 s	Wound healing	[142]
Polyurethane (PU)	TiO_2_/CNFs	Cellulose nano crystals (CNC) and polydimethylsiloxane (PDMS)	Above 50 °C	Biomedical application	[165]
PLLA	PDA/CuNPs	-	48.2 °C	Infectious bone defect treatment	[166]

### 5.2. Non-Antibacterial Applications of Photothermal Electrospun Fibers

Photothermal electrospinning technology demonstrates considerable promise in non-antibacterial applications, primarily encompassing two major domains: tumor therapy (Table 2) and tissue regeneration (Table 3).

Cancer remains one of the most formidable diseases confronting humanity in recent decades. According to data from the National Cancer Institute in 2018, approximately 609,640 cancer-related deaths were projected, with an estimated 1,735,350 new cases diagnosed with this lethal disease [167,168,169]. Conventional cancer treatments, including surgery, radiotherapy (RT), and chemotherapy, often face limitations such as incomplete tumor eradication and systemic side effects [170]. Consequently, researchers have been actively pursuing more targeted and effective therapeutic approaches. In recent years, PTT has emerged as a novel treatment modality, garnering significant attention in oncology due to its unique advantages in thermal-induced physical ablation, circumvention of drug resistance, and post-treatment immunomodulation [170,171,172].

**Figure 7:**
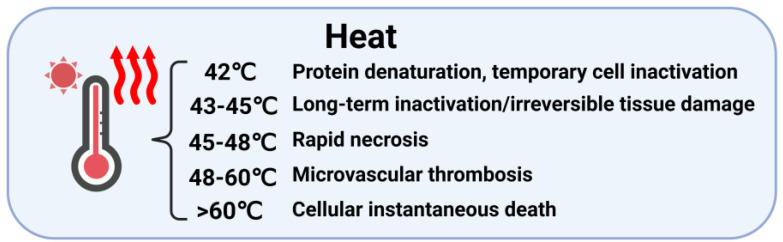
Temperature-dependent killing mechanism of PTT on tumor cells. Created in BioRender. Wang, K. (2025) https://BioRender.com/6lgu69u.

The antitumor mechanism of PTT primarily relies on thermal ablation exceeding 42 °C. At 42 °C, protein denaturation and temporary cellular inactivation occur. With increasing temperature, progressive biological effects manifest: long-term inactivation/irreversible tissue damage (43–45 °C), rapid necrosis (45–48 °C), and microvascular thrombosis (48–60 °C). When temperatures surpass 60 °C, instantaneous cell death occurs due to protein denaturation and plasma membrane disruption (Figure 7) [16,83,172].

Electrospinning provides a facile approach to incorporating PTAs into fibrous materials. The implantation of such materials at tumor sites followed by NIR irradiation enables localized photothermal ablation. For example, Shao et al. [173] fabricated Bi_2_Se_3_/PLLA membranes by co-electrospinning Bi_2_Se_3_ with PLLA, achieving nearly 100% cancer cell inactivation after 5 min of 808 nm laser irradiation (0.5 W/cm^2^) in both in vitro and in vivo experiments. Sui et al. [113] compared blended PDA/PLLA nanofibers with coated PDA@PLLA electrospun nanofibers, with the latter demonstrating superior tumor ablation efficacy. Additionally, Li et al. [102] developed ultrathin BP nanosheets (BP@SF) using silk fibroin (SF) as an exfoliating agent via ultrasound-assisted liquid-phase exfoliation, which were subsequently incorporated into SF/PLGA/BP@SF photothermal electrospun membranes that effectively killed HeGp2 cancer cells through photothermal effects.

However, current research consensus suggests that complete eradication of solid tumors using PTT alone, particularly after a single treatment course, remains challenging. Combination strategies integrating PTT with other modalities (e.g., PDT, chemotherapy, and immunotherapy) may capitalize on the strengths of each approach while mitigating individual limitations, thereby generating additive or even synergistic therapeutic outcomes, which has been demonstrated in the previous section [83].

The most extensively investigated combination in photothermal electrospinning involves PTT–chemotherapy integration. PTT-mediated degradation of the tumor stromal barrier facilitates drug diffusion, coupled with light-controlled drug release that minimizes systemic toxicity. By incorporating chemotherapeutic agents into fibrous delivery systems, enhanced antitumor efficacy can be achieved through photothermal effects. Doxorubicin (DOX) and paclitaxel (PTX) represent the most commonly employed model drugs, frequently co-loaded with PTAs in electrospun fibers to enable NIR-responsive controlled release. Zhang et al. [174] demonstrated that PLLA nanofibers co-loaded with DOX and multi-walled carbon nanotubes (MWCNTs) as PTAs exhibited both temperature elevation and DOX burst release under NIR irradiation, attributable to PLLA’s relatively low glass transition temperature (T_g_) and thermally enhanced drug diffusion. Other chemotherapeutic agents, including dihydromyricetin, trametinib, docetaxel, bortezomib, and all-trans retinoic acid, have also been reported in similar systems.

Accurate on-demand drug release concisely within tumor tissues poses a major research obstacle. Therefore, the integration of multi-stimuli responsiveness under physiological conditions has become a research focus in advanced fiber fabrication [175]. A recent study developed a novel bifunctional biomaterial combining PTT–chemotherapy with hemostatic properties for preventing post-surgical recurrence and metastasis of skin cancer. The PLGA/MXene/DOX nanofiber@chitosan (PMD@CS) aerogel was prepared by freeze-drying a suspension containing chitosan (CS) and electrospun PLGA/MXene/DOX nanofibers. Under NIR irradiation, MXene generated hyperthermia for tumor ablation while simultaneously altering the PLGA nanofiber structure to accelerate DOX molecular motion. Concurrently, protonation of CS’s -NH_2_ groups in the acidic tumor microenvironment enhanced hydrophilicity and promoted DOX release, collectively enabling temperature–pH dual-responsive drug release behavior. Notably, bleeding represents a critical factor in post-surgical tumor recurrence and metastasis. The inherent hemostatic properties of CS, attributed to its functional groups (e.g., strongly positive-charged amino groups that attract negatively charged phospholipid layers on platelets and erythrocytes), facilitated rapid aggregation and coagulation [176].

Most current studies on PTT–chemotherapy via electrospinning exploit the acidic tumor microenvironment (compared with that in normal tissues) for more precise drug delivery, in addition to PTA-mediated photothermal ablation and NIR-responsive drug release. Interestingly, a recent photothermal electrospinning study considered the dynamic redox environmental changes following tumor resection surgery—initial oxidative stress increase, followed by transition to a more reductive environment. The authors loaded PTX and gold nanorods (AuNRs) onto redox-responsive glutathione-extended polyurethane urea (PolyCEGS) electrospun membranes, achieving integrated redox/NIR light-responsive chemotherapy and photothermal effects. This system enabled controlled local PTX release at tumor sites post-surgery, combining drug and PTT effects to prevent recurrence while enhancing cytotoxicity [177].

In addition to chemotherapy, photodynamic therapy (PDT) employs photosensitizers to generate reactive oxygen species (ROS) under specific light wavelengths and oxygen, often combined with PTT for physical–chemical synergistic antitumor effects. In Yang et al.’s study, CS/PEO-CuSe composite fibrous membranes exhibited both excellent photothermal and photodynamic properties through CuSe incorporation [178]. Beyond CuSe’s inherent antiproliferative effects, 0.4 wt.% CS/PEO-CuSe nanofibrous membranes reached 56 °C and generated sufficient ROS under 1064 nm irradiation to kill both cancer cells and bacteria [178]. However, the efficacy of PDT is oxygen-dependent, which limits the effectiveness of PTT–PDT in deep-seated or metastatic tumors, making it more suitable for superficial malignancies such as skin cancer. In the currently published literature on photothermal electrospun systems, the application of PTT–PDT in oncology remains underexplored, likely due to its constrained antitumor performance.

In the aforementioned applications, PTT typically achieves local temperatures exceeding 45 °C, effectively killing bacteria and tumor cells but potentially damaging surrounding healthy tissues and limiting utility in tissue engineering and regenerative medicine. Consequently, mild photothermal therapy (MPTT, 37–42 °C) has attracted growing research interest. Recent studies suggest that mild heat can promote tissue repair and regeneration through potential mechanisms involving accelerated blood flow, enhanced nutrient exchange, heat shock protein (HSP) induction, and ion channel activation [179,180,181]. The mild photothermal effects of PTA-incorporated electrospun materials have thus opened new avenues in regenerative medicine, including bone tissue engineering, neural repair, and skin wound healing.

Ma et al. [182] developed PCL/MoS_2_ composite electrospun membranes that delivered mild thermal stimulation (40.5 ± 0.5 °C) via NIR irradiation to promote tibial bone defect regeneration in rats. Building upon MPPT applications, strategies incorporating multifunctional integration and bone tissue biomimetics can significantly enhance both regenerative efficacy and material biocompatibility. For instance, Li et al. [104] proposed a photothermal double-layer biomimetic periosteum with neurovascular coupling via electrospinning, demonstrating superior bone regeneration performance in both in vitro and in vivo experiments compared with the control groups. Structurally, this biomimetic periosteum comprised a conventional electrospun outer layer preventing soft tissue invasion and an aligned nanofibrous inner layer facilitating cell recruitment and angiogenesis. Functionally, neodymium-doped photothermal whitlockite (Nd@WH) played a pivotal role: sustained Mg^2+^ release effectively promoted nerve growth factor (NGF) and vascular endothelial growth factor (VEGF) upregulation, while Ca^2+^ and PO_4_^3−^ release combined with photothermal osteogenesis (40.5 ± 0.5 °C) synergistically enhanced bone regeneration. In another study, Zhang et al. [102] functionalized BP nanosheet-incorporated aligned electrospun fibers with Apt19S to selectively recruit mesenchymal stem cells (MSCs) to injury sites for bone repair initiation, while depositing antibiotic-loaded phase change material microparticles on scaffold surfaces for temperature-triggered bacterial elimination.

Beyond bone regeneration, recent studies have revealed the remarkable potential of PTA–electrospun membrane combinations in neural and skin tissue repair. Hu et al. [183] fabricated gold polydopamine blackspheres (AuPBs) with excellent NIR absorption and photothermal conversion properties through dopamine and chloroauric acid redox reactions, which were then loaded onto PLLA-SF electrospun membranes (PSPFs) to create neural scaffolds (PSPF@AuPBs). Implantation at sciatic nerve injury sites demonstrated that PDA alleviated local inflammation and promoted VEGF expression and neovascularization. Under NIR irradiation, periodic TRPV1 ion channel opening in Schwann cells induced Ca^2+^ influx and upregulated brain-derived neurotrophic factor (BDNF) and NGF expression, facilitating nerve regeneration and functional recovery. In Xiao et al. [103]’s study, ultrasmall CuS@BSA nanoparticles with mild photothermal conversion capability were shown to induce MSC differentiation into fibroblasts, thereby promoting skin regeneration. Although CuS@BSA was not directly incorporated into electrospun membranes in this study, both in vitro (where decreases in the MSC nuclear-to-cytoplasmic ratio indicated fibroblast differentiation under 42 °C thermal stimulation) and in vivo experiments demonstrated superior wound closure effects, providing valuable insights for the future development of nanoparticle-based photothermal electrospun systems.

**Table 2:** Photothermal electrospun fibers for anticancer treatment.

Polymers	Photothermal Agents	Other Additives	Ref.
PLA	Multi-walled carbon nanotubes (MWCNTs)	DOX	[174]
PVA/CS	MoS_2_	DOX	[184]
Gelatin/PCL	CuS	Dihydromyricetin	[185]
Redox-responsive Glutathione-extended polyurethane urea derivative (PolyCEGS)	AuNRs	PTX	[177]
PCL/PDLLA	Copper silicate hollow microspheres (CSO HMSs)	Trametinib	[186]
PCL	PDA	DOX	[114]
PCFs/PBS/LA@ZIF-8	GNRs	DOX	[187]
Alginate-dopamine/PVA (Outer and inner layers) PLA/PCL (Middle layer)	PDA NPs	Docetaxel	[112]
PLA/PCL/Gelatin	Prussian blue (PB)	Hydroxychloroquine sulfate (HCQ)	[188]
PLGA/CS aerogel	MXene	DOX	[176]
Polydioxanone (PDO)	PDA NPs	Bortezomib (BTZ)	[189]
PLGA	Au NRs	DOX	[190]
PCL/Gelatin	PDA NPs	DOX	[191]
Poly (tetramethylene ether) glycol based-polyurethane (PTMG-PU) (core)/chitosan (shell)	GO/Au NRs	PTX	[110]
PCL	Hydroxylated multi-walled carbon nanotubes (MWCNTsOH)	All-trans retinoic acid (ATRA)	[192]
CS/PVA	Indocyanine green (ICG)	DOX	[193]
PCL	gold nanocage (AuNC)	DOX/phase-changeable fatty acid	[111]
PCL/PLGA	Pyrrole	DOX	[194]
PCL	Polypyrrole (PPy)	PTX	[195]
PCL	PDA	DOX	[114]
Polyacrylonitrile (PAN)/polymethyl methacrylate (PMMA)	PCNFs	DOX	[196]
PLGA/PLA-b-PEG	PEGylated gold nanorods (PEG-GNRs)	-	[197]
Gelatin/PCL	Polyaniline nanoparticles	-	[198]
PCL	GO	-	[122]
SF/PLGA	SF-modified BP nanosheets (BP@SF)	-	[199]
PLLA	PDA NPs	-	[113]
PCL	Polypyrrole hollow fibers (PPy-HFs)	-	[200]
PLLA	Bi_2_Se_3_	-	[178]
CS/PEO	CuSe NPs	-	[173]
Gelatin/PCL	DOX-Cu_9_S_5_@mSiO_2_	-	[201]

**Table 3:** Photothermal electrospun fibers for tissue regeneration.

Polymers	Photothermal Agents	Other Additives	Temperature Reached	Application Field	Ref.
PLA	CuS@BSA	-	42 °C	Skin tissue regeneration	[103]
PLLA/SF	Gold-polydopamine (PDA) blackspheres (AuPBs)	-	35 °C	Neural tissue regeneration	[99]
PCL	Ti_3_C_2_T_x_ MXene	-	-	Neural tissue regeneration	[183]
PVP/PLA	GO	Urolithin A (UA)	52.2 °C over 2 min 30 s	Bone tissue regeneration	[129]
PCL	Nd@WH	-	40.5 ± 0.5 °C after 1 min about 40 °C	Bone tissue regeneration	[104]
PCL	MoS_2_	-	40.5 ± 0.5 °C	Guided bone regeneration	[182]
PCL	BP NSs	Apt19S Lauric acid and stearic acid	around 40 °C	Bone fracture repair	[102]

## 6. Challenges of Practical Applications

Despite remarkable advancements in photothermal electrospinning technology within the biomedical field in recent years, several critical challenges persist in translating these innovations into practical applications.

### 6.1. Material Selection

Current inorganic photothermal materials (e.g., gold nanoparticles and carbon-based materials) exhibit excellent photothermal conversion efficiency, yet concerns remain regarding their long-term biosafety and biodegradability, with potential accumulation in vital organs leading to chronic toxicity [202,203]. While organic photothermal agents demonstrate superior biocompatibility, they frequently suffer from photobleaching and inadequate thermal stability [204,205]. Although well-dispersed or rigid nano-PTAs may exhibit nanoreinforcement effects on electrospun fibers under specific conditions, PTA incorporation often negatively impacts fiber properties. For instance, (i) the agglomeration of PTAs (e.g., AuNPs and MoS_2_) can induce stress concentration within fibers, reducing tensile strength, and (ii) hydrophobic PTAs (e.g., carbon nanotubes) tend to undergo phase separation when blended with hydrophilic polymers (e.g., PVA), increasing fiber brittleness.

### 6.2. Industrial Production and Clinical Translation

The transition from laboratory- to industrial-scale-production faces significant technical barriers.

The foremost challenge shared across the electrospinning field is poor batch-to-batch reproducibility due to the complexity of the processing parameters [206,207,208,209]. For coaxial/emulsion/multi-needle electrospinning techniques, maintaining stable structures requires an evaluation of more intricate fluid dynamic systems. Although post-modification processes can circumvent some parameter optimization issues, multi-step processing inevitably reduces cost-effectiveness [209,210,211,212,213].

Moreover, limitations in the production rate of current techniques significantly hinder scale-up manufacturing. Conventional electrospinning technology typically achieves a production rate of 0.01–1 g/h by fiber weight or 1.0–5.0 mL/h by flow rate, which falls significantly short of industrial-scale production requirements [210,214].

To date, photothermal electrospinning technology and its efficacy assessment have been confined to laboratory-scale studies, with no reported preclinical or clinical trials. The clinical translation of this approach faces significant hurdles due to its numerous technical constraints.

Traditional sterilization methods, such as high-temperature and high-pressure treatments and γ irradiation, may compromise the structural integrity of electrospun fibers and the functionality of PTAs or bioactive materials [215,216].

Furthermore, a comprehensive evaluation of the long-term toxicological properties of PTAs is absolutely critical prior to human application, which mainly depend on their composition and degeneration pathways. For CuS, their degradation products (Cu^2+^) may exceed the hepatic metabolic threshold (>0.1 mg/kg/day), which would undoubtedly cause organ-specific accumulation and severe health risks [217,218]. For MXene, the main mechanisms of their toxicity to animal cells are oxidative stress and mechanical damage to cell membranes caused by sharp nanosheet edges [219]. Carbon nanotubes (CNTs), including the single-walled (SWCNTs) and multi-walled (MWCNTs) variants, have attracted significant concerns regarding their potential health impacts in recent years. Current studies demonstrate that CNTs can adversely affect the immune system, inducing pulmonary inflammation, asthmatic symptoms, and even interstitial fibrosis. Murine experiments have revealed the genotoxic potential of MWCNTs. Certain types of MWCNTs have been classified as possible human carcinogens [220,221].

### 6.3. Therapeutic Efficacy

While the localized administration of photothermal electrospun fibers enables more precise temperature control compared with conventional PTT approaches, heterogeneity in tissue thermal conductivity may still lead to uneven heat distribution, resulting in either insufficient treatment of target areas or thermal damage to healthy tissues. Beyond employing combination therapies to reduce thermal dose requirements, future solutions may include (i) loading thermal feedback materials (e.g., phase-change materials) for autonomous temperature regulation; (ii) designing heterostructures in photothermal agents or electrospun fibers to improve thermal conduction uniformity; (iii) implementing closed-loop control systems for real-time monitoring and dynamic adjustment; (iv) optimizing irradiation techniques for spatial selectivity to spare healthy tissues; and (v) utilizing molecular modifications or microenvironment-responsive release for targeted delivery.

Additionally, current photothermal electrospun materials are primarily suitable for superficial tissue applications, with treatment depth limited by two factors: (i) the penetration depth of NIR light in biological tissues is typically limited to approximately 1 cm and (ii) electrospun fiber materials are predominantly administered as patches or dressings. The substitution of traditional NIR-I (650–900 nm) agents with NIR-II (1000–1350 nm) photothermal materials constitutes a straightforward yet effective approach, leveraging NIR-II’s superior tissue penetration depth (3–5 cm) and diminished scattering properties. Upconversion nanoparticles (UCNPs) enable the transformation of deeply penetrating 980 nm irradiation into localized shorter-wavelength emission, which can subsequently activate proximal photothermal agents. Consequently, the co-encapsulation of UCNPs with photothermal agents enhances their efficacy in deep tissue applications. Additionally, processing electrospun fibers into implantable or injectable formats could significantly broaden their clinical applicability beyond surface tissues [14,76,222].

## 7. Conclusions and Outlook

Photothermal electrospinning technology has established a unique therapeutic platform for biomedical applications through the integration of photothermal agents into electrospun fibers. This approach not only preserves the advantageous characteristics of electrospun materials, including high surface area and structural tunability, but also endows them with precise photothermal responsiveness. Current research has confirmed that the judicious selection of photothermal agent types, loading methods, and fiber architectures can achieve diverse therapeutic objectives, ranging from high-temperature ablation of bacteria and cancer cells to mild thermotherapy for tissue regeneration.

Practical applications of this technology remain constrained by several formidable challenges, including material selection, industrial-scale production, clinical translation, and therapeutic efficacy, which hinder further advancement of photothermal electrospun materials and represent critical directions for future research. The existing specific solutions have been provided in the preceding text.

As materials science and biomedicine continue to converge, photothermal electro-spinning is poised to emerge as a pivotal platform for next-generation precision medicine, offering innovative solutions for disease treatment. Based on comprehensive examination and research practice, this review identifies several promising future directions for photo-thermal electrospinning in biomedical applications: (i) development of innovative multi-functional integrated materials; (ii) implementation of AI-assisted parameter optimization; (iii) design of patient-specific personalized therapies; (iv) exploration of novel applications in biomedical fields; and (v) facilitation of translational research from preclinical studies to clinical trials.

## Data Availability

Not applicable.
